# Imaging-associated stress causes divergent phase transitions of RNA-binding proteins in the *Caenorhabditis elegans* germ line

**DOI:** 10.1093/g3journal/jkac172

**Published:** 2022-07-08

**Authors:** Mohamed T Elaswad, Chloe Munderloh, Brooklynne M Watkins, Katherine G Sharp, Elizabeth Breton, Jennifer A Schisa

**Affiliations:** Biochemistry, Cell and Molecular Biology Program, Central Michigan University, Mt. Pleasant, MI 48859, USA; Department of Biology, Central Michigan University, Mt. Pleasant, MI 48859, USA; Department of Biology, Central Michigan University, Mt. Pleasant, MI 48859, USA; Present address Department of Molecular and Human Genetics, Baylor College of Medicine, Houston, TX 77030, USA; Biochemistry, Cell and Molecular Biology Program, Central Michigan University, Mt. Pleasant, MI 48859, USA; Department of Biology, Central Michigan University, Mt. Pleasant, MI 48859, USA; Department of Biology, Central Michigan University, Mt. Pleasant, MI 48859, USA; Present address Department of Biological Sciences, University of Pittsburgh, Pittsburgh, PA 15260, USA; Department of Biology, Central Michigan University, Mt. Pleasant, MI 48859, USA; Present address Van Andel Research Institute, Grand Rapids, MI 49503, USA; Biochemistry, Cell and Molecular Biology Program, Central Michigan University, Mt. Pleasant, MI 48859, USA; Department of Biology, Central Michigan University, Mt. Pleasant, MI 48859, USA

**Keywords:** stress, phase transition, RNP granule, RNA-binding protein, condensation, oogenesis, live imaging

## Abstract

One emerging paradigm of cellular organization of RNA and RNA-binding proteins is the formation of membraneless organelles. Examples of membraneless organelles include several types of ribonucleoprotein granules that form via phase separation. A variety of intracellular pH changes and posttranslational modifications, as well as extracellular stresses, can stimulate the condensation of proteins into granules. For example, the assembly of stress granules induced by oxidative stress, osmotic stress, and heat stress has been well characterized in a variety of somatic cell types. In the germ line, similar stress-induced condensation of proteins occurs; however, less is known about the role of phase separation during gamete production. Researchers who study phase transitions often make use of fluorescent reporters to study the dynamics of RNA-binding proteins during live cell imaging. In this report, we demonstrate that common conditions of live-imaging *Caenorhabditis elegans* can cause an inadvertent stress and trigger phase transitions of RNA-binding proteins. We show that this imaging-associated stress stimulates decondensation of multiple germ granule proteins and condensation of several P-body proteins. Proteins within larger ribonucleoprotein granules in meiotically arrested oocytes do not appear to be as sensitive to the stress as proteins in diakinesis oocytes of young hermaphrodites, with the exception of the germ granule protein PGL-1. Our results have important methodological implications for all researchers using live-cell imaging techniques. The data also suggest that the RNA-binding proteins within large ribonucleoprotein granules of arrested oocytes may have distinct phases, which we characterize in our companion article.

## Introduction

Phase separation is an emerging principle of cellular organization. Among the well-studied membraneless organelles that assemble via liquid–liquid phase separation are ribonucleoprotein (RNP) granules composed of mRNA and RNA-binding proteins. Types of RNP granules include processing bodies and stress granules in an array of cell types and germ granules in the germ line (Corbet and Parker 2019). Many RNA-binding proteins in RNP granules can undergo reversible phase transitions among diffuse, liquid, and gel-like phases. Transitions among these phases can function as an adaptation to environmental and intracellular changes and regulate mRNA metabolism (Alberti and Carra 2018). In a variety of somatic cells, stress granules are induced by unfavorable environmental conditions such as oxidative stress, temperature changes, hypoxia, and osmotic stress to promote cellular homeostasis (Ivanov *et al.* 2019). Similarly, condensates can be induced by stress in the germ line; examples include stress granules in mouse spermatocytes, reticulated sponge bodies and large U-bodies in *Drosophila* egg chambers, and large, hybrid RNP granules in *Caenorhabditis elegans* oocytes (Schisa 2019).

Proper regulation of phase transitions is critical, as dysregulation of RNP granules is associated with disease states such as cancer, cardiovascular disease, and neurodegenerative diseases (Riggs *et al.* 2020; Davis *et al.* 2022). For example, in ALS (amyotrophic lateral sclerosis) mutations, the FUS protein phase separates into abnormal neuronal granules, and ectopic aggregates of FMRpolyG protein in ovarian stromal cells are associated with Fragile-X-associated primary ovarian insufficiency (Ganion 1991; Buijsen *et al.* 2016; Friedman-Gohas *et al.* 2020). While a growing number of in vivo studies have started to build on in vitro observations that demonstrate a role for multivalent interactions in driving the condensation and decondensation of proteins, the precise regulation and function of phase transitions in oogenesis is not well understood.

The *Caenorhabditis* germ line provides an excellent model to study questions of RNA-binding protein condensation (Schisa *et al.* 2001; Jud *et al.* 2008; Wood *et al.* 2016). In a young *C. elegans* hermaphrodite, oocytes undergo meiotic maturation every 23 min; however, within a few days of adulthood, the sperm become depleted and meiosis arrests an extended time (McCarter *et al.* 1999). In the arrested oocytes, several RNA-binding proteins condense into large RNP granules that are up to 20 times larger than a typical cytoplasmic P granule in oocytes (Schisa *et al.* 2001). The condensation is reversible if sperm are resupplied via mating, or major sperm protein is injected (Schisa *et al.* 2001; Jud *et al.* 2008). Several mRNAs and diverse types of RNA-binding proteins are detected in the large RNP granules including P-granule proteins, P-body proteins, stress granule proteins, and other RNA-binding proteins associated with translational regulation such as the KH-domain protein MEX-3 and PUF domain protein PUF-5 (Schisa *et al.* 2001; Jud *et al.* 2008; Noble *et al.* 2008; Hubstenberger *et al.* 2013). Based on their composition, the large, hybrid RNP granules are hypothesized to regulate mRNA metabolism during extended delays in the fertilization of oocytes.

In this study, we uncover a live imaging-associated stress that causes divergent phase transitions of RNA-binding proteins in oocytes. While PGL-1 and GLH-1 decondense in diakinesis oocytes of young hermaphrodites in response to extended imaging, MEX-3, CGH-1, and CAR-1 undergo condensation into granules. We show that these proteins are generally not as sensitive to imaging-associated stress when they are already condensed into large RNP granules in arrested oocytes. The differential stress responses may suggest distinct phases of RNA-binding proteins within large RNP granules. Finally, our results have important implications for all researchers using live cell-imaging methods, especially those studying phase transitions.

## Methods

### Strains and maintenance

All worms were grown on nematode growth media using standard conditions at 20°C (Brenner 1974) unless specified. Strains used include: DG4269 mex-3(tn1753[gfp::3xflag::mex3]), OD61 ItIs41[pAA5; pie-1::GFP-TEV-Stag::CAR-1; unc-119(+)], JH3644 *fog-2(g71)* V; meg-3(ax4320)[meg-3::mCherry]X, CL2070 dvIs70 [hsp-16.2::GFP + rol-6(su1006)], SJ4005 zcIs4 [hsp-4::GFP] V, LD1171 IdIs4 [gcs-1p::GFP + rol-6(su1006)], OH16024 daf-16(ot971[daf-16::GFP]), JH3269 pgl-1(ax3122[pgl-1::gfp]), DUP64 *glh-1(sam24[glh-1::GFP::3xFLAG])*, and JH1985 *unc-119(ed3); axIs1436[pCG33* pie-1prom: LAP::CGH-1]. Some strains were crossed into CB4108 *fog-2(q71)* as noted. Worms were synchronized using the hypochlorite bleach method.

### Microscopy and image analysis

Worms were picked onto slides made with 2% agarose pads and paralyzed using 6.25 mM levamisole. To image diakinesis oocytes of young hermaphrodites, synchronized 1 day post-L4-stage worms were used. To image meiotically arrested oocytes in *fog-2* strains, L4 females were separated from males and imaged 1 or 2dpL4 as indicated in figure legends.

Images were collected using a Leica compound fluorescence microscope (low magnification images of whole worms in Fig. 4) or a Nikon A1R laser scanning confocal microscope (all other images). All images for a given strain were collected using identical levels and settings. The number of granules in oocytes and total integrated density (intensity of fluorescence in granules) was determined using ImageJ particle analysis. In Fig. 4, the threshold to categorize dispersed PGL-1 was determined by the lowest fluorescence intensity in granules seen in the control 0–10 min worms. The threshold for condensed CAR-1 was determined to be >3× higher intensity than in control 0-10 min. worms.

### Imaging using oxidative stress and ER stress reporters

The positive control for *gcs-1*::GFP was incubating young adults at 34°C for 3.5 hr and recovering at 20°C for 1 hr before imaging (An and Blackwell 2003). The positive control for *hsp-16.2*::GFP was 34°C for 2 hr and recovering at 20°C for 12 hr before imaging (Strayer *et al.* 2003). The positive control for *hsp*-4::GFP was 8 hr at 30°C before imaging (Bischof *et al.* 2008). Controls were compared to worms imaged within <25 or 60–90 min after being prepared. GFP levels in images were qualitatively scored.

### Statistical analysis

Sample sizes were determined using G*Power 3.1 for power analyses; all experiments were blinded and done at least in triplicate. Data are presented as mean ± SEM unless otherwise indicated. Statistical analyses were performed on GraphPad Prism 9.1, and specific tests are noted in figure legends. *P*-values <0.05 were considered statistically significant.

## Results and discussion

### Imaging conditions can inadvertently induce stress and phase transitions

**Fig. 1.**
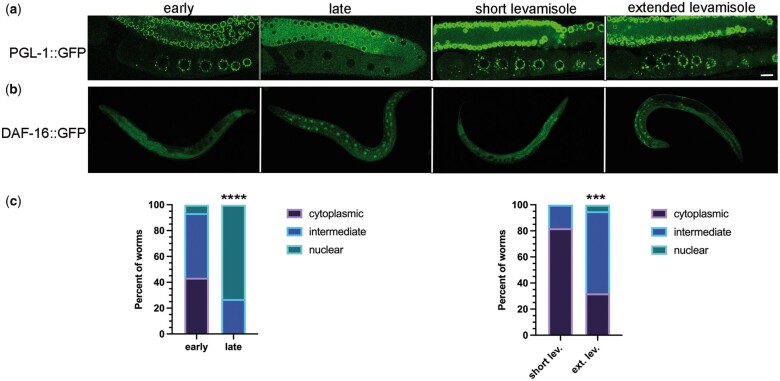
Imaging conditions can inadvertently induce stress. a) Subcellular localization of PGL-1::GFP in the germ line: early during imaging process (within 25 min) or after extended time imaging (60 min); and after short (15 min) or extended (60 min) exposure to 6.25 mM levamisole prior to mounting worms on a slide. Proximal oocytes are oriented on the bottom left in all images. Scale bar is 10 µm. b) Subcellular localization of DAF-16::GFP in young hermaphrodites: imaging was done early (within 25 min) or late (after 60–90 min) of slides being prepared and after short (15 min) or extended (60 min) exposure to 6.25 mM levamisole. c) Graphs show the percentage of DAF-16::GFP worms with cytoplasmic distribution, intermediate distribution, or nuclear localization. Statistical significance was determined using the Fisher’s exact test. **** indicates *P* < 0.0001 and *** indicates *P* < 0.001. *n* = 15 (early), 16 (late), 28 (short), and 19 (extended).

The regulation of RNA-binding protein phase transitions in the germ line is not well understood. While investigating candidate regulators of the RGG domain-protein PGL-1 in the adult germ line, we noticed unanticipated dispersal of PGL-1 during imaging of control RNAi-treated worms. Early in the imaging session, PGL-1 was detected in punctate P granules throughout the adult germ line as expected (Strome and Wood 1982); however, over extended time (referred to as late), PGL-1 became dispersed in oocytes and the distal core, appearing at increased levels throughout the cytosol and decreased levels in punctate granules (Fig. 1a). To determine if our imaging conditions were inadvertently inducing a stress response, we used the DAF-16::GFP reporter strain (Aghayeva *et al.* 2020). The DAF-16 FOXO (forkhead box class O) transcription factor translocates from the cytoplasm to the nucleus in response to unfavorable conditions such as heat stress, oxidative stress, starvation, or when cellular repair is needed (Oh *et al.* 2005; Henderson *et al.* 2006). We categorized the subcellular localization of DAF-16 as cytoplasmic, nuclear, or intermediate (mix of cytoplasmic and nuclear) (Oh *et al.* 2005). We found that DAF-16 was cytoplasmic or intermediate in the large majority of worms imaged within 25 min of slides being prepared (worms picked into 6.25 mM levamisole on a 2% agarose pad and coverslip added), and DAF-16 was nuclear in less than 10% of worms (Fig. 1, b and c). For the purpose of a contrasting experiment we chose a much later time interval; imaging was conducted 60–90 min after slides were prepared (for convenience we refer to these conditions as extended imaging). In contrast to the early-imaged worms, 73% of worms that were scored late had nuclear localization of DAF-16, and none were cytoplasmic. Based on these results, we consider extended imaging conditions an imaging-associated stress.

Levamisole is a paralytic agent frequently used as an anesthetic to mount worms for imaging. While one study showed that DAF-16 remains cytoplasmic after worms are exposed to 1 mm levamisole and recovered for 30 min (Manjarrez and Mailler 2020), exposure to levamisole can affect autophagy and result in increased numbers of GFP::LGG-1 puncta in somatic cells (Zhang *et al.* 2015). We therefore asked if extended exposure to levamisole is sufficient to induce PGL-1 decondensation or DAF-16 translocation to nuclei. We incubated PGL-1::GFP worms in a drop of 50 µl of 6.25 mM levamisole for 15 or 60 min in a humidity chamber, prior to placing them on an agarose pad and imaging within 15 min. We observed punctate PGL-1 granules in both samples, with no sign of the dispersed PGL-1 observed in late-imaged worms (Fig. 1a). In a large majority of DAF-16::GFP worms incubated a short time in levamisole, we observed cytoplasmic DAF-16, with only 18% having an intermediate phenotype of partial nuclear translocation (Fig. 1, b and c). In contrast, 63% of worms incubated an extended amount of time in levamisole had intermediate DAF-16 translocation, and in 5% of worms, DAF-16 was nuclear. Because the “extended levamisole” worms exhibited a weaker DAF-16 stress response than the late-imaged worms, we conclude levamisole contributes to stress during imaging, but is not the only source of stress. Our results suggest that while levamisole can contribute to a mild DAF-16 stress response, additional aspects of extended imaging conditions underlie the decondensation of PGL-1 out of granules.

### Imaging-associated stress causes divergent phase transitions of RNA-binding proteins

**Fig. 2.**
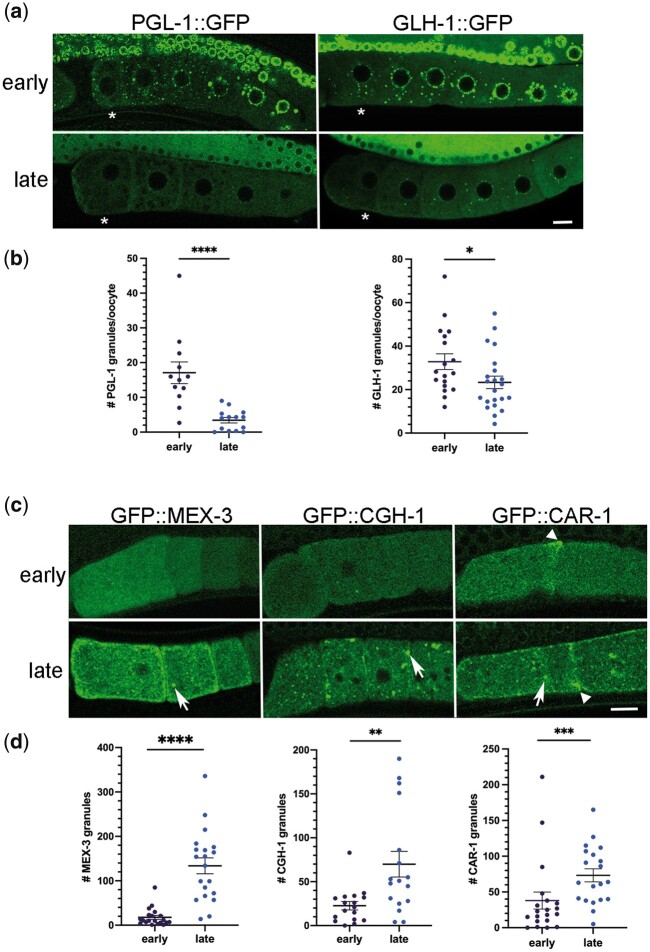
Imaging-associated stress causes distinct phase transitions among RNA-binding proteins. a) Micrographs of GFP-tagged P granule protein reporter strains, PGL-1::GFP and GLH-1::GFP. Top row: distribution of GFP in germ line early during imaging; bottom row: distribution of GFP in germ line after extended imaging (late) with strongest phenotypes shown. Asterisk marks the most proximal oocyte in each germ line. Smaller distal pachytene nuclei are visible at top of each image. Scale bar is 10 µm. b) Graphs showing the number of GFP granules in a single Z-slice of proximal oocytes (see *Methods*). Statistical significance was determined using the Mann–Whitney test. **** indicates *P* < 0.0001; * indicates *P* < 0.05. *n* = 16–26. c) Micrographs of GFP-tagged RNA-binding protein reporter strains (MEX-3, CGH-1, and CAR-1) in most proximal oocytes. Top row: distribution of GFP early during imaging; Bottom row: distribution of GFP after extended imaging (late) with strongest phenotypes shown. Arrows indicate ectopic granules; arrowheads indicate cortical enrichment. Scale bar is 10 µm. d) Graphs showing the number of GFP granules in a single Z-slice of proximal oocytes. Statistical significance was determined using the Mann–Whitney test. **** indicates *P* < 0.0001, *** indicates *P* < 0.001, and ** indicates *P* < 0.01. *n* = 17–20. Error bars indicate mean ± SEM.

To determine the extent to which imaging-associated stress affects phase transitions of RNA-binding proteins in diakinesis oocytes of young hermaphrodites, we first quantitated the effects on two P-granule proteins using the same conditions as for DAF-16. As expected for PGL-1, given our initial observations during RNAi experiments (Fig. 1), we observed significantly fewer granules of PGL-1::GFP in oocytes and fewer granules in the distal nuclei, late during imaging (Fig. 2, a and b). We also observed significant decondensation of GFP::GLH-1 in oocytes and the distal nuclei, although the dispersal of protein out of granules was not as penetrant as with PGL-1 (Fig. 2, a and b). We conclude extended imaging can induce the decondensation of multiple P-granule proteins.

We next asked if imaging-associated stress affects three RNA-binding proteins that are normally dispersed throughout oocytes of young hermaphrodites. MEX-3 is a KH-domain protein that localizes to P granules in embryos but is mostly diffuse throughout the cytosol of oocytes (Draper *et al.* 1996). In response to extended imaging the majority of GFP::MEX-3 protein appeared to remain decondensed throughout the cytoplasm; however, we detected a variable but significant increase in the number of small GFP::MEX-3 granules enriched cortically in oocytes (Fig. 2, c and d). For phenotypes detected predominantly at the oocyte cortex, we chose to include cortical confocal slices that typically do not include the nuclei (Fig. 2c). CGH-1 and CAR-1 are orthologs of the P-body proteins Me31B/RCK and Trailerhitch/RAP55/Lsm14 (Schisa 2012). Both proteins are detected in P granules and throughout the cytosol of oocytes (Navarro *et al.* 2001; Boag *et al.* 2005). After extended imaging, the number of GFP::CGH-1 granules was variably increased; in a small number of worms, more than 7 times as many granules were detected compared to the control (Fig. 2, c and d). Imaging-associated stress also resulted in increased numbers of GFP::CAR-1 granules that were enriched at the nuclear envelope, as well as enriched along the cortical membrane (Fig. 2, c and d). We also observed CAR-1 enrichment in nonspherical aggregates, distinct from the spherical granules, at the cortex in a subset of control and stressed worms (Fig. 1c, arrowhead). Taken together, we conclude an imaging-associated stress can induce opposing RNA-binding protein phase transitions in diakinesis oocytes of young hermaphrodites. MEX-3, CGH-1, and CAR-1 proteins undergo condensation into granules, opposite of the decondensation observed for PGL-1 and GLH-1.

**Fig. 3.**
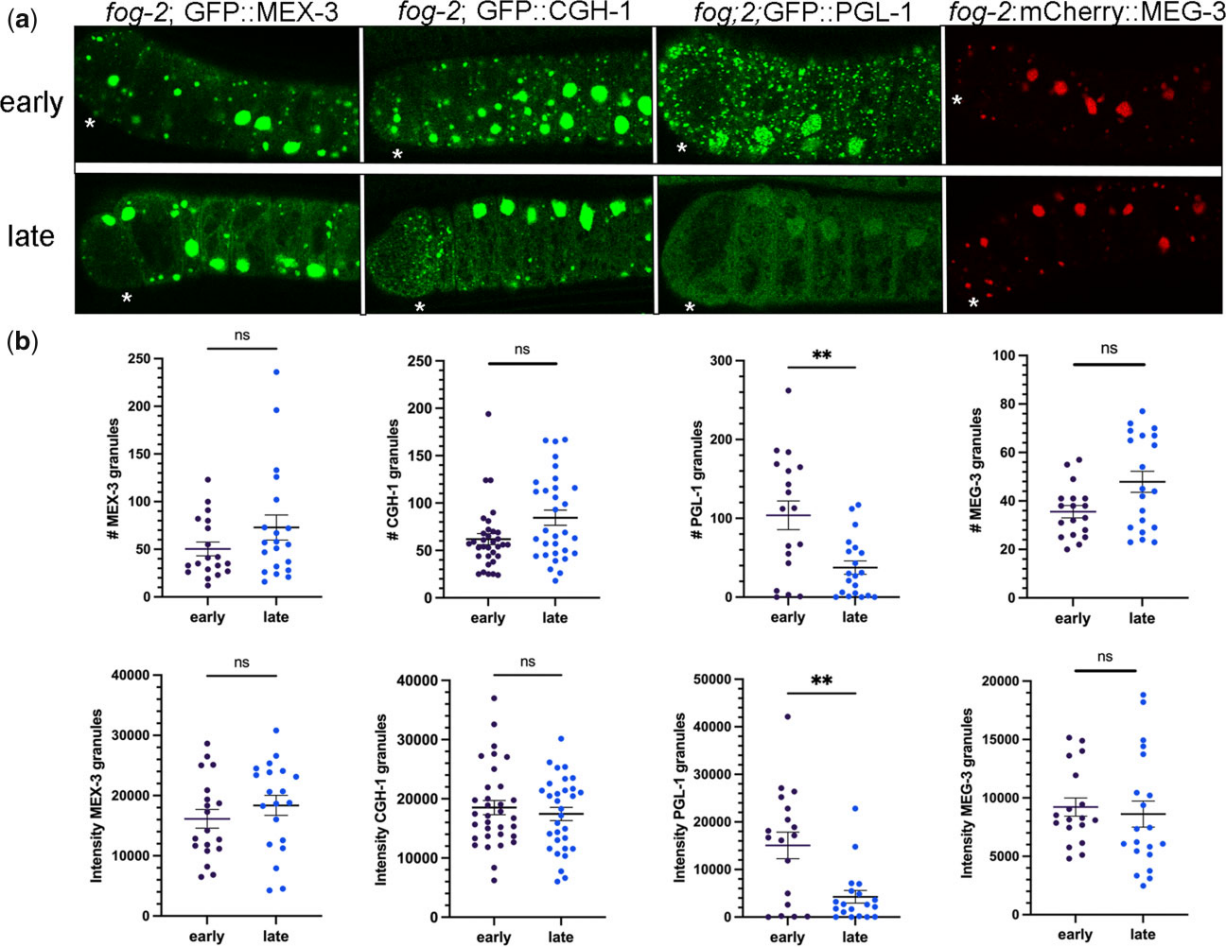
Imaging-associated stress preferentially affects PGL-1 within large RNP granules of meiotically arrested oocytes. a) Micrographs of GFP-tagged RNA-binding proteins (MEX-3, CGH-1, PGL-1, MEG-3) in a *fog-2* background. Top row: distribution of GFP in arrested oocytes early during imaging. Bottom row: distribution of GFP in arrested oocytes after extended imaging (late). Asterisk marks the most proximal oocyte in each germ line. Scale bar is 10 µm. b) Graphs showing either the number of GFP granules or the integrated density of GFP in granules in a single Z-slice of proximal oocytes (see *Methods*). Statistical significance was determined using the Mann–Whitney test. ** indicates *P* < 0.01 and ns indicates not significant. *n* = 18–33. Error bars indicate mean ± SEM.

The dynamics of proteins within granules can decrease as the size of RNP granules increases (Hubstenberger *et al.* 2013). Moreover, the properties and functions of RNA-binding proteins can be disrupted upon phase transitions. For example, in *Drosophila* egg chambers in the absence of the E3 ubiquitin ligase HECW, the Me31B RNA-binding protein transitions from small granules in a liquid phase to larger granules in a gel-like phase, and translational repression of target mRNAs is deregulated (Fajner *et al.* 2021). Since slower dynamics and increased stability can occur as RNP granules increase in size, we asked if imaging-associated stress affects phase transitions of four RNA-binding proteins that condense into large RNP granules in meiotically arrested oocytes of *fog-2* females. In early-imaged oocytes, we observed large cortical granules of GFP::MEX-3 and GFP::CGH-1 as expected (Schisa *et al.* 2001; Jud *et al.* 2008; Noble *et al.* 2008). After extended imaging, we did not detect any significant changes in the number of MEX-3 or CGH-1 granules or in the intensity of fluorescence in granules, suggesting no major change in condensation (Fig. 3, a and b). In contrast, PGL-1 granules were significantly reduced in number and intensity after extended imaging, compared to the “early” control where a heterogeneous mix of small and very bright, large granules was detected as expected (Fig. 3, a and b; Jud *et al.* 2008; Noble *et al.* 2008). Thus, we conclude imaging-associated stress induces decondensation of PGL-1 out of large granules in arrested oocytes, similar to its effect on the small P granules in oocytes of young hermaphrodites. Lastly, we examined the effect of extended imaging on MEG-3. MEG-3 is an intrinsically disordered protein normally detected at low levels in oocytes and strongly localized to P granules in embryos (Wang *et al.* 2014). We found that during extended meiotic arrest in a *fog-2* background, MEG-3 underwent a dramatic phase transition and condensed into large granules in oocytes, as has been previously reported (Fig. 3a;Putnam *et al.* 2019). We detected no significant changes in MEG-3 granule number or intensity after extended imaging (Fig. 3, a and b). Taken together, these data suggest that while MEX-3 and CGH-1 are sensitive to imaging-associated stress in diakinesis oocytes and condense into granules, if the proteins are already condensed into large granules in arrested oocytes, they are more stable during imaging conditions. Our data are also consistent with a model where the MEX-3, CGH-1, and MEG-3 phases of large RNP granules are less liquid-like than PGL-1, since PGL-1 is sensitive to imaging-associated stress in both diakinesis and arrested oocytes. Our companion article tests this model of distinct phases using multiple functional assays (Elaswad *et al.* 2022).

### Extended imaging does not appear to induce oxidative stress or ER stress

**Fig. 4.**
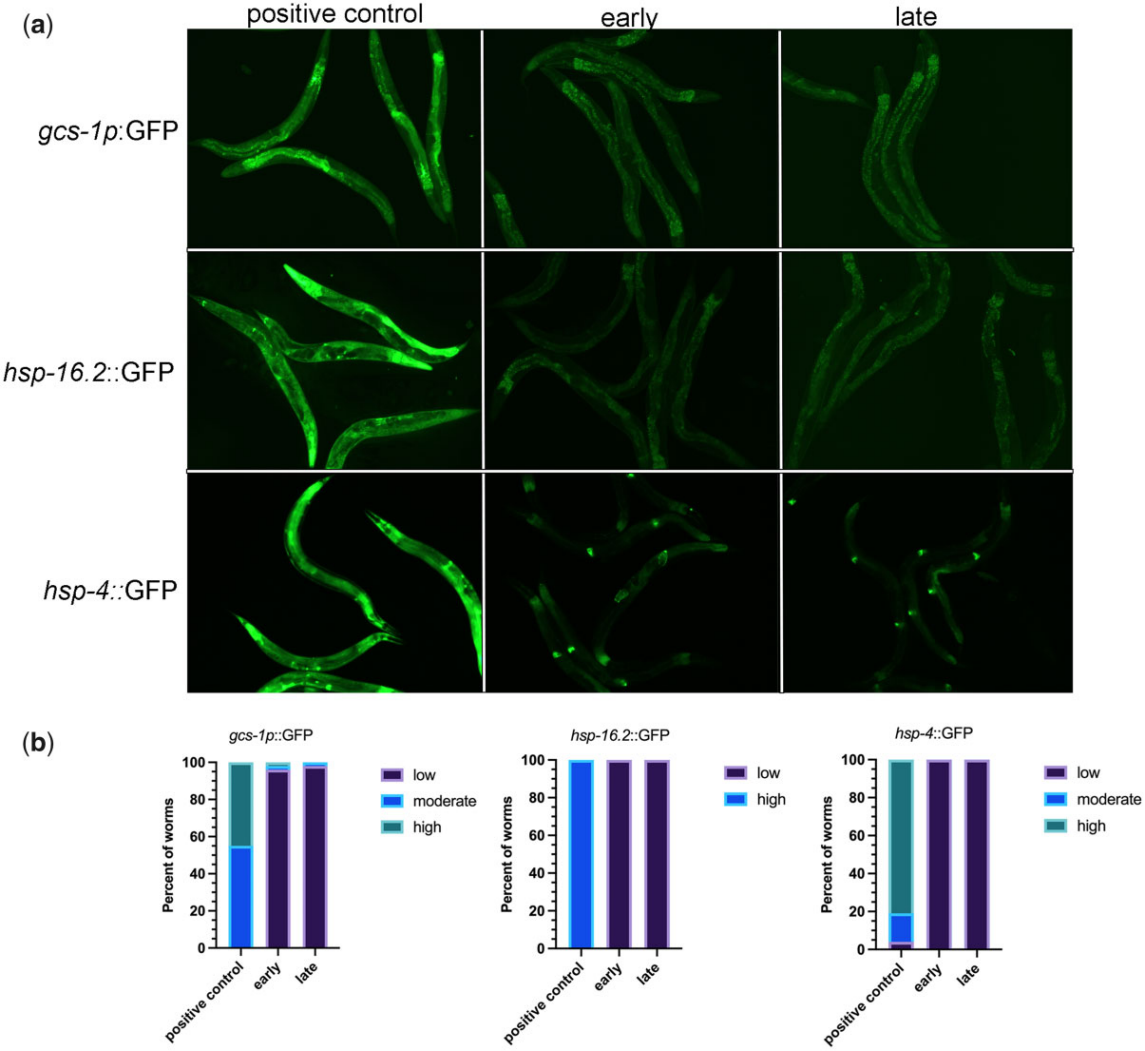
Extended imaging does not appear to induce oxidative or ER stress. a) Micrographs of *gcs-1*::GFP, *hsp-16.2*::GFP, and *hsp-4*::GFP young adult worms either early during imaging or after extended imaging. The positive control for each was heat stress (see *Methods*). b) Graphs show the percentage of worms with low, moderate, or high levels of expression of GFP. No significant differences were detected between early and late-imaged worms in any of the reporter strains. *n* = 27–54.

Once we established extended imaging can modulate phase transitions, we wanted to identify the type of stress induced by the imaging conditions. We used two reporter strains to ask if oxidative stress is induced: *gcs-1p*::gfp and *hsp-16.2*::gfp. GCS-1 (gamma-glutamyl-cysteine synthetase heavy gene) is a detoxification gene that is induced in the intestine in response to oxidative stress, including heat-induced increases in intracellular reactive oxygen species (An and Blackwell 2003; Wang *et al.* 2010). HSP-16.2 is a small heat shock protein that is also induced by oxidative stress (Link *et al.* 1999). In the positive control, heat-stressed *gcs-1p*::gfp worms, we observed moderate or high GFP expression in 100% of the worms (Fig. 4a). In contrast, in oocytes assayed early during imaging without heat stress, moderate or high levels of GFP were detected in only 4% of worms, and the GFP expression did not significantly increase after extended imaging (Fig. 4b). In these experiments, levels of expression were assessed qualitatively using reference images. In the positive control, heat-stressed *hsp-16.2*::gfp worms, high levels of GFP expression were seen in 100% of worms. In contrast, low levels of expression were observed in worms imaged either early or after extended imaging (Fig. 4, a and b). We also asked if ER stress is induced during imaging by using the *hsp-4*::gfp reporter. HSP-4 is the immunoglobulin heavy chain-binding protein (BiP) homolog, and a reporter for the unfolded protein response that prepares cells for expansion of the ER (Shen *et al.* 2001; Kapulkin *et al.* 2005; Walter and Ron 2011). In the positive control, after 8 hr of 30°C heat stress, we observed moderate or high GFP levels in 96% of worms as expected (Bischof *et al.* 2008). In contrast, 100% of worms imaged early had low levels of GFP and 98% of worms maintained low GFP levels after extended imaging (Fig. 4b). Overall, these results suggest that the oxidative, ER stress, and heat stress pathways are not stimulated at high levels by imaging-associated stress, and a different type of stress is induced by extended imaging. Prior studies report that a variety of stresses including osmotic stress, starvation, and anoxia can trigger the condensation of RNA-binding proteins in the germ line (Jud *et al.* 2008; Huelgas-Morales *et al.* 2016; Davis *et al.* 2017). We speculate that a hypoxic stress may occur during extended imaging due to prolonged coverslip exposure; however, we were not able to find a suitable reporter to test this hypothesis in adults.

### Imaging conditions can induce a rapid stress response

**Fig. 5.**
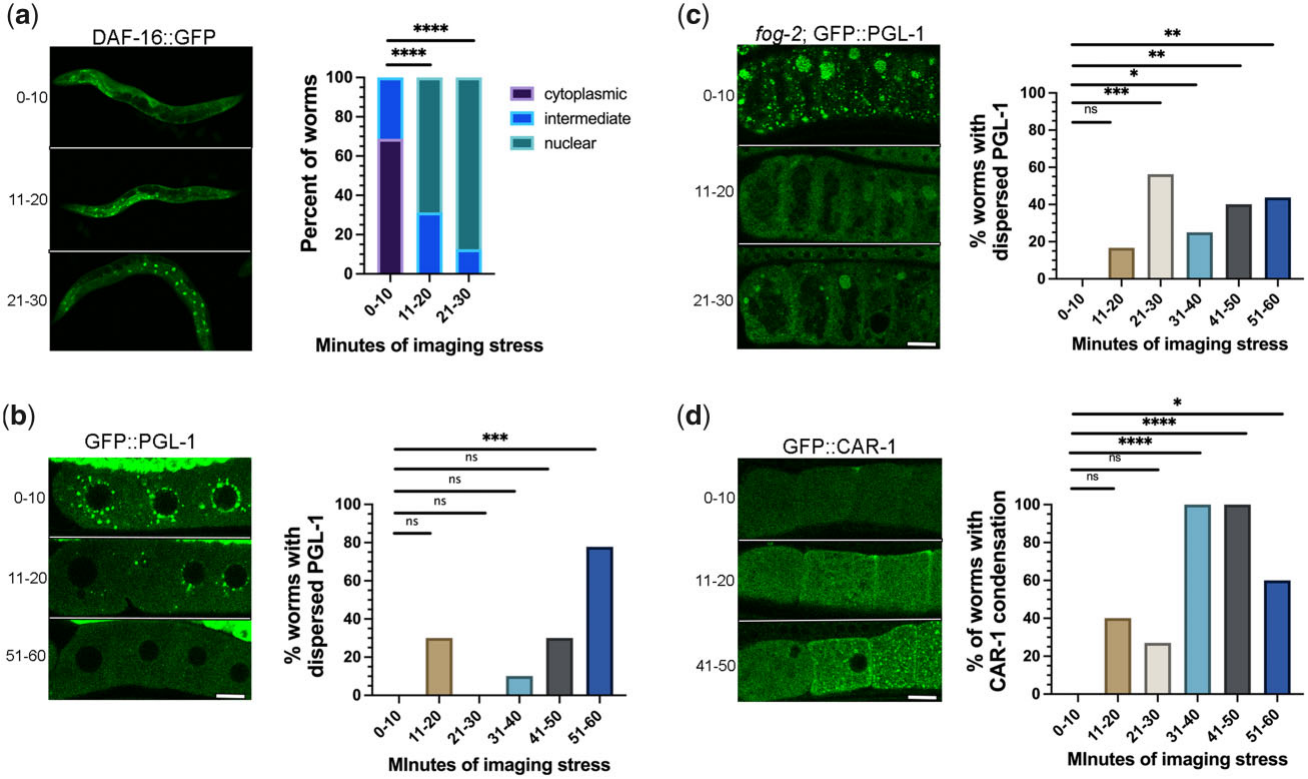
Imaging conditions can quickly trigger stress and modulate phase transitions. a–d) Time course of the effects of imaging-associated stress on DAF-16 nuclear translocation, and condensation of PGL-1 and CAR-1. For each reporter, micrographs of GFP-tagged strains are shown on the left with proximal oocytes oriented to the left, and graphs show the percentage of worms with nuclear translocation (DAF-16) or phase transitions (PGL-1, CAR-1). Whole hermaphrodites are shown in (a); diakinesis oocytes in young hermaphrodites are shown in (b) and (d); and arrested oocytes are in (b). All scale bars are 10 µm. See *Methods* and text for the description of thresholds used to categorize phase transitions. Statistical significance was determined using the Kruskal–Wallis test. **** indicates *P* < 0.0001, *** indicates *P* < 0.001, ** indicates *P* < 0.01, * indicates *P* < 0.05, and ns indicates not significant. *n* = 9–15.

Having established that an imaging-associated stress can differentially trigger condensation or decondensation of RNA-binding proteins, we asked how quickly the stress occurs while imaging. We first examined DAF-16::GFP worms that were prepared prior to imaging for a maximum of 10, 20, or 30 min. In the majority of 0–10-min worms, DAF-16 was cytoplasmic, and none had strong nuclear localization (Fig. 5a). In contrast after 11–20 min, DAF-16 was nuclear in 69% of worms and intermediate in the remaining 31% of worms (Fig. 5a). In the 21–30 min worms, DAF-16 was nuclear in >80% of worms. These time course data indicate that some level of stress occurs within 11–20 min of worms being prepared for imaging and is relevant for all researchers using live-imaging methods to consider.

We next asked if RNA-binding protein phase transitions are induced as quickly as DAF-16 nuclear translocation. We first examined PGL-1 and found that, in 30% of 11–20-min worms, PGL-1 was at least partially decondensed out of granules, compared to the 0–10-min worms, which had bright PGL-1 granules as expected (Fig. 5b). We categorized PGL-1 as dispersed when the PGL-1 intensity in granules was below the lowest intensity observed in the 0–10-min worms (see *Methods*). The decondensation of PGL-1 was variable at all time points, and not statistically significant until the 51–60-min worms; however, it was clear that at least modest decondensation occurs in a subset of worms within the same rapid time frame as DAF-16 nuclear translocation. We next assayed PGL-1 in arrested oocytes after 0–10 min of imaging and observed a mix of small and large PGL-1 granules at the oocyte cortex as expected (Fig. 5c). PGL-1 appeared dispersed in 17% of worms at 11–20 min, and within 21–30 min of imaging, we observed a significant increase in the percentage of worms with dispersed PGL-1 (Fig. 5c). At later time points, we continued to observe the dispersal of PGL-1; however, the penetrance of the response was variable (Fig. 5c). These data suggest that PGL-1 is not more stable when condensed into large granules in arrested oocytes; in fact, it appears more sensitive to stress than when in small granules of diakinesis oocytes (Fig. 5b). Lastly, we examined the response of CAR-1 in diakinesis oocytes. In the 0–10-min worms, CAR-1 was mostly diffuse throughout the cytoplasm as expected (Fig. 5d). After 11–20 min, CAR-1 condensation was observed in 40% of worms; condensation was defined as worms having >3× as many CAR-1 granules at the cortex as the largest number of granules in 0–10-min worms (Fig. 5d). At the three time points beyond 30 min, CAR-1 was condensed in a significant % of worms (60–100%; Fig. 5d). Based on these observations, we conclude that while imaging-associated stress induces changes to both DAF-16 translocation and RNA-binding protein condensation, the effects on RNA-binding proteins are more variable. Nonetheless, inadvertent phase transitions of decondensation or condensation can occur within 11–20 min of imaging, which is fairly rapid, especially if researchers routinely prepare their worms at a location distant from their microscopy facility.

## Conclusions

The number of researchers studying in vivo phase-separated RNA-binding proteins has grown tremendously over the past decade and resulted in a paradigm shift in our understanding of cellular organization. Here, we describe a potential pitfall of live-cell imaging using fluorescent reporter strains to study phase transitions. Our results demonstrate that liquid-like proteins, such as PGL-1, are extremely sensitive to decondensation during imaging conditions, dispersing out of granules in as few as 11–20 min. Moreover, other RNA-binding proteins are sensitive to ectopic condensation during imaging. Our results showing inadvertent phase transitions of germ line RNA-binding proteins in the *C. elegans* model system seem likely to have broad applicability to live-cell imaging in all *in vivo* systems. Careful controls of imaging conditions are clearly critical to avoid inadvertent phase transitions. Furthermore, because the disruption of phase transitions can alter the function of RNA-binding proteins, our results are pertinent not only when studying phase transitions, but also for all physiological assays.

## Data availability

All strains are available at the *Caenorhabditis* Genetics Center or on request from the Schisa lab or other *C. elegans* labs. The authors affirm that all data necessary to confirm the conclusions in this article are included in the article, figures, and tables.

## References

[jkac172-B1] Aghayeva U , Bhattacharya A, Hobert O. A panel of fluorophore-tagged daf-16 alleles. MicroPubl Biol. 2020. doi:10.17912/micropub.biology.000210. PMC7252392 32550509

[jkac172-B2] Alberti S , Carra S. Quality control of membraneless organelles. J Mol Biol. 2018;430(23):4711–4729. 29758260 10.1016/j.jmb.2018.05.013

[jkac172-B3] An JH , Blackwell TK. SKN-1 links C. elegans mesendodermal specification to a conserved oxidative stress response. Genes Dev. 2003;17(15):1882–1893. 12869585 10.1101/gad.1107803 PMC196237

[jkac172-B4] Bischof LJ , Kao CY, Los FC, Gonzalez MR, Shen Z, Briggs SP, van der Goot FG, Aroian RV. Activation of the unfolded protein response is required for defenses against bacterial pore-forming toxin in vivo. PLoS Pathog. 2008;4(10):e1000176. 18846208 10.1371/journal.ppat.1000176 PMC2553261

[jkac172-B5] Boag PR , Nakamura A, Blackwell TK. A conserved RNA-protein complex component involved in physiological germline apoptosis regulation in C. elegans. Development. 2005;132(22):4975–4986. 16221731 10.1242/dev.02060

[jkac172-B6] Brenner S. The genetics of Caenorhabditis elegans. Genetics. 1974;77(1):71–94. 4366476 10.1093/genetics/77.1.71 PMC1213120

[jkac172-B7] Buijsen RA , Visser JA, Kramer P, Severijnen EA, Gearing M, Charlet-Berguerand N, Sherman SL, Berman RF, Willemsen R, Hukema RK. Presence of inclusions positive for polyglycine containing protein, FMRpolyG, indicates that repeat-associated non-AUG translation plays a role in fragile X-associated primary ovarian insufficiency. Hum Reprod. 2016;31(1):158–168. 26537920 10.1093/humrep/dev280 PMC4677964

[jkac172-B8] Corbet GA , Parker R. RNP granule formation: lessons from P-bodies and stress granules. Cold Spring Harb Symp Quant Biol. 2019;84:203–215. 32482896 10.1101/sqb.2019.84.040329

[jkac172-B9] Davis M , Montalbano A, Wood MP, Schisa JA. Biphasic adaptation to osmotic stress in the *C. elegans* germ line. Am J Physiol Cell Physiol. 2017;312(6):C741–C748. 28381521 10.1152/ajpcell.00364.2016 PMC5494592

[jkac172-B10] Davis RB , Moosa MM, Banerjee PR. Ectopic biomolecular phase transitions: fusion proteins in cancer pathologies. Trends Cell Biol. 2022:S0962-8924(22)00077-0. doi: 10.1016/j.tcb.2022.03.005. PMC9288518 35484036

[jkac172-B11] Draper BW , Mello CC, Bowerman B, Hardin J, Priess JR. MEX-3 is a KH domain protein that regulates blastomere identity in early C. elegans embryos. Cell. 1996;87(2):205–216. 8861905 10.1016/s0092-8674(00)81339-2

[jkac172-B12] Elaswad MT , Watkins BM, Sharp KG, Munderloh C, Schisa JA. Large RNP granules in C. elegansoocytes have distinct phases of RNA binding proteins. 2022;12(9):jkac173. doi: 10.1093/g3journal/jkac173. PMC9434171 35816006

[jkac172-B13] Fajner V , Giavazzi F, Sala S, Oldani A, Martini E, Napoletano F, Parazzoli D, Cesare G, Cerbino R, Maspero E, et al Hecw controls oogenesis and neuronal homeostasis by promoting the liquid state of ribonucleoprotein particles. Nat Commun. 2021;12(1):5488. 34531401 10.1038/s41467-021-25809-8 PMC8446043

[jkac172-B14] Friedman-Gohas M , Elizur SE, Dratviman-Storobinsky O, Aizer A, Haas J, Raanani H, Orvieto R, Cohen Y. FMRpolyG accumulates in FMR1 premutation granulosa cells. J Ovarian Res. 2020;13(1):22. doi:10.1186/s13048-020-00623-w. 32101156 PMC7045455

[jkac172-B15] Ganion LR. Cytoplasmic distribution of poly(A)-containing RNA in developing Necturus maculosus oocytes with reference to annulate lamellae. Anat Rec. 1991;230(2):218–224. 1714257 10.1002/ar.1092300209

[jkac172-B16] Henderson ST , Bonafe M, Johnson TE. daf-16 protects the nematode Caenorhabditis elegans during food deprivation. J Gerontol A Biol Sci Med Sci. 2006;61(5):444–460. 16720740 10.1093/gerona/61.5.444

[jkac172-B17] Hubstenberger A , Noble SL, Cameron C, Evans TC. Translation repressors, an RNA helicase, and developmental cues control RNP phase transitions during early development. Dev Cell. 2013;27(2):161–173. 24176641 10.1016/j.devcel.2013.09.024 PMC3869996

[jkac172-B18] Huelgas-Morales G , Silva-García CG, Salinas LS, Greenstein D, Navarro RE. The stress granule RNA-binding protein TIAR-1 protects female germ cells from heat shock in Caenorhabditis elegans. G3 (Bethesda). 2016;6(4):1031–1047. 26865701 10.1534/g3.115.026815 PMC4825639

[jkac172-B19] Ivanov P , Kedersha N, Anderson P. Stress granules and processing bodies in translational control. Cold Spring Harb Perspect Biol. 2019;11(5):a032813. 30082464 10.1101/cshperspect.a032813 PMC6496347

[jkac172-B20] Jud MC , Czerwinski MJ, Wood MP, Young RA, Gallo CM, Bickel JS, Petty EL, Mason JM, Little BA, Padilla PA, et al Large P body-like RNPs form in C. elegans oocytes in response to arrested ovulation, heat shock, osmotic stress, and anoxia and are regulated by the major sperm protein pathway. Dev Biol. 2008;318(1):38–51. 18439994 10.1016/j.ydbio.2008.02.059 PMC2442018

[jkac172-B21] Kapulkin WJ , Hiester BG, Link CD. Compensatory regulation among ER chaperones in C. elegans. FEBS Lett. 2005;579(14):3063–3068. 15907843 10.1016/j.febslet.2005.04.062

[jkac172-B22] Link CD , Cypser JR, Johnson CJ, Johnson TE. Direct observation of stress response in Caenorhabditis elegans using a reporter transgene. Cell Stress Chaper. 1999;4(4):235–242. 10.1379/1466-1268(1999)004<0235:doosri>2.3.co;2 PMC312938 10590837

[jkac172-B23] Manjarrez JR , Mailler R. Stress and timing associated with Caenorhabditis elegans immobilization methods. Heliyon. 2020;6(7):e04263. 32671240 10.1016/j.heliyon.2020.e04263 PMC7339059

[jkac172-B24] McCarter J , Bartlett B, Dang T, Schedl T. On the control of oocyte meiotic maturation and ovulation in Caenorhabditis elegans. Dev Biol. 1999;205(1):111–128. 9882501 10.1006/dbio.1998.9109

[jkac172-B25] Navarro RE , Shim EY, Kohara Y, Singson A, Blackwell TK. cgh-1, a conserved predicted RNA helicase required for gametogenesis and protection from physiological germline apoptosis in C. elegans. Development. 2001;128(17):3221–3232. 11546739 10.1242/dev.128.17.3221

[jkac172-B26] Noble SL , Allen BL, Goh LK, Nordick K, Evans TC. Maternal mRNAs are regulated by diverse P body-related mRNP granules during early Caenorhabditis elegans development. J Cell Biol. 2008;182(3):559–572. 18695046 10.1083/jcb.200802128 PMC2500140

[jkac172-B27] Oh SW , Mukhopadhyay A, Svrzikapa N, Jiang F, Davis RJ, Tissenbaum HA. JNK regulates lifespan in Caenorhabditis elegans by modulating nuclear translocation of forkhead transcription factor/DAF-16. Proc Natl Acad Sci U S A. 2005;102(12):4494–4499. 15767565 10.1073/pnas.0500749102 PMC555525

[jkac172-B28] Putnam A , Cassani M, Smith J, Seydoux G. A gel phase promotes condensation of liquid P granules in Caenorhabditis elegans embryos. Nat Struct Mol Biol. 2019;26(3):220–226. 30833787 10.1038/s41594-019-0193-2 PMC6668929

[jkac172-B29] Riggs CL , Kedersha N, Ivanov P, Anderson P. Mammalian stress granules and P bodies at a glance. J Cell Sci. 2020;133(16):jcs242487. 32873715 10.1242/jcs.242487 PMC10679417

[jkac172-B30] Schisa JA. New insights into the regulation of RNP granule assembly in oocytes. Int Rev Cell Mol Biol. 2012;295:233–289. 22449492 10.1016/B978-0-12-394306-4.00013-7 PMC3875216

[jkac172-B31] Schisa JA. Germ cell responses to stress: the role of RNP granules. Front Cell Dev Biol. 2019;7:220. doi:10.3389/fcell.2019.00220. 31632971 PMC6780003

[jkac172-B32] Schisa JA , Pitt JN, Priess JR. Analysis of RNA associated with P granules in germ cells of C. elegans adults. Development. 2001;128(8):1287–1298. 11262230 10.1242/dev.128.8.1287

[jkac172-B33] Shen X , Ellis RE, Lee K, Liu CY, Yang K, Solomon A, Yoshida H, Morimoto R, Kurnit DM, Mori K, et al Complementary signaling pathways regulate the unfolded protein response and are required for C. elegans development. Cell. 2001;107(7):893–903. 11779465 10.1016/s0092-8674(01)00612-2

[jkac172-B34] Strayer A , Wu Z, Christen Y, Link CD, Luo Y. Expression of the small heat-shock protein Hsp16-2 in Caenorhabditis elegans is suppressed by Ginkgo biloba extract EGb 761. FASEB J. 2003;17(15):2305–2307. 14525938 10.1096/fj.03-0376fje

[jkac172-B35] Strome S , Wood WB. Immunofluorescence visualization of germ-line-specific cytoplasmic granules in embryos, larvae, and adults of Caenorhabditis elegans. Proc Natl Acad Sci U S A. 1982;79(5):1558–1562. 7041123 10.1073/pnas.79.5.1558 PMC346014

[jkac172-B36] Walter P , Ron D. The unfolded protein response: from stress pathway to homeostatic regulation. Science. 2011;334(6059):1081–1086. 22116877 10.1126/science.1209038

[jkac172-B37] Wang J , Robida-Stubbs S, Tullet JM, Rual JF, Vidal M, Blackwell TK. RNAi screening implicates a SKN-1-dependent transcriptional response in stress resistance and longevity deriving from translation inhibition. PLoS Genet. 2010;6(8):e1001048. doi:10.1371/journal.pgen.1001048. 20700440 PMC2916858

[jkac172-B38] Wang JT , Smith J, Chen BC, Schmidt H, Rasoloson D, Paix A, Lambrus BG, Calidas D, Betzig E, Seydoux G. Regulation of RNA granule dynamics by phosphorylation of serine-rich, intrinsically disordered proteins in C. elegans. eLife. 2014;3:e04591. doi:10.7554/eLife.04591. 25535836 PMC4296509

[jkac172-B39] Wood MP , Hollis A, Severance AL, Karrick ML, Schisa JA. RNAi screen identifies novel regulators of RNP granules in the Caenorhabditis elegans germ line. G3 (Bethesda). 2016;6(8):2643–2654. 27317775 10.1534/g3.116.031559 PMC4978917

[jkac172-B40] Zhang H , Chang JT, Guo B, Hansen M, Jia K, Kovacs AL, Kumsta C, Lapierre LR, Legouis R, Lin L, et al Guidelines for monitoring autophagy in Caenorhabditis elegans. Autophagy. 2015;11(1):9–27. 25569839 10.1080/15548627.2014.1003478 PMC4502811

